# Polycythemia Vera and Cardiovascular Disease: A Mini Review

**DOI:** 10.14789/ejmj.JMJ25-0065-R

**Published:** 2026-03-13

**Authors:** MONE MUKAI, TADAO AIKAWA, YUYA MATSUE, TOHRU MINAMINO

**Affiliations:** 1 Faculty of Medicine, Juntendo University, Tokyo, Japan; 1 Faculty of Medicine, Juntendo University, Tokyo, Japan; 2 Department of Cardiovascular Biology and Medicine, Juntendo University Graduate School of Medicine, Tokyo, Japan; 2 Department of Cardiovascular Biology and Medicine, Juntendo University Graduate School of Medicine, Tokyo, Japan

**Keywords:** polycythemia vera, cardiovascular disease, ropeginterferon α-2b, cardio-oncology, cardiotoxicity

## Abstract

Polycythemia vera (PV) is a chronic myeloproliferative neoplasm characterized by clonal erythrocytosis driven by JAK2 mutations, affecting more than 95% of patients. Morbidity and mortality in PV are dominated by cardiovascular complications―particularly arterial and venous thrombosis. Modern treatment approaches, including therapeutic phlebotomy, low-dose aspirin, and cytoreductive therapies (hydroxyurea, interferon-α formulations, and ruxolitinib), have reduced blood cell counts and thrombotic risk, yet concerns about treatment-associated cardiovascular toxicity have emerged. Recent cardio-oncology guidelines emphasize structured monitoring for cancer therapy-related cardiovascular toxicity. This mini review summarizes the cardiovascular burden of PV, therapeutic strategies to mitigate risk, and emerging perspectives on cardiotoxicity―including a recently reported case of reversible left ventricular dysfunction associated with ropeginterferon α-2b.

## Introduction

Polycythemia vera (PV) is a chronic myeloproliferative neoplasm characterized by clonal erythrocytosis due to constitutive activation of the Janus kinase (JAK)/signal transducer and activator of transcription (STAT) pathway, predominantly through the JAK2 V617F mutation^1)^. Approximately 65,000 individuals in the United States have the disease, which has a median age of diagnosis of 65 years and is slightly more prevalent in men (male-to- female ratio, 1.3-1.6)^2)^. In addition to erythrocytosis, PV often presents thrombocytosis, leukocytosis, splenomegaly, and aquagenic pruritus^3)^. The major clinical concerns of PV are thrombotic events, hemorrhage, and progression to myelofibrosis or acute myeloid leukemia^3, 4)^.

The JAK2 sequence variant is also found in the general population without PV. In a population- based cohort from Denmark, the JAK2 V617F mutation was identified in about 3% of 19,958 individuals aged ≥ 20 years^5)^. In this context, the presence of the mutation appears consistent with clonal hematopoiesis of indeterminate potential. Importantly, carriers of this sequence variant demonstrated a markedly increased likelihood of developing coronary artery disease compared with noncarriers, with a pooled hazard ratio of 12.0 (95% confidence interval, 3.8-38.4) derived from four case-control analyses including 4,726 coronary heart disease cases and 3,529 controls^6)^.

### Thrombotic and hemorrhagic risks in PV

Thrombotic and hemorrhagic complications are a major cause of illness and death in patients with PV. At the time of diagnosis, approximately 16% and 7% of patients have arterial and venous thromboses, respectively^3)^. After diagnosis, an additional 12% and 9% of patients develop arterial and venous thromboses, respectively^2)^. Retrospective data from over 1,500 patients with PV indicate that thromboembolism and heart failure have been reported as the leading causes of death following acute leukemia and second malignancies^3)^. The risk of thrombosis increases with age, a prior history of thrombosis, an elevated hematocrit, leukocytosis (>11×10^9^/L), and a high JAK2 V617F allele burden (≥ 50%)^2, 7)^. Although low-dose aspirin and anticoagulant therapy are used to prevent thromboembolism^1)^, hemorrhagic events are frequently observed in patients with PV receiving these treatments (1.4-6.8% patients/year)^8)^.

### Strategies to reduce thrombotic risk

Phlebotomy to maintain a hematocrit <45% is the cornerstone of PV management^1, 2)^. The CYTO-PV randomized trial demonstrated a 3.9-fold reduction in cardiovascular events when hematocrit was controlled below 45% compared to targets between 45-50%^4)^. Low-dose aspirin (75-100 mg daily) is universally recommended for patients with PV unless contraindicated^2)^. The ECLAP trial found a 60% reduction in major cardiovascular events with aspirin versus placebo among PV patients, though there was a nonsignificant increase in major bleeding (1.2% vs. 0.8%)^9)^. Management of traditional cardiovascular risk factors, such as hypertension, diabetes, hyperlipidemia, and smoking cessation, is also recommended.

Cytoreductive therapy is indicated for high-risk patients (age ≥ 60 or with a history of thrombosis) or those with persistent symptoms, in order to reduce thrombotic risk and control hematocrit levels below 45%. Hydroxyurea remains the first-line treatment for PV; however, interferon (IFN) formulations (ropeginterferon α-2b and pegylated IFN-α2a) and the JAK1/JAK2 inhibitor ruxolitinib are alternative options for patients who do not achieve an adequate reduction in hematocrit levels or experience severe side effects such as pruritus from hydroxyurea^1, 2, 10-13)^.

### Cardiac dysfunction related to PV treatments

Cancer therapy-related cardiac dysfunction (CTRCD) is the broad spectrum of left ventricular systolic dysfunction that occurs secondary to cancer therapy, including chemotherapy, targeted therapy, immune therapy, or radiation^14-16)^. The 2022 European Society of Cardiology (ESC) cardio-oncology guidelines define CTRCD as meeting any of the following criteria: (1) a decline in left ventricular ejection fraction (LVEF) ≥ 10% to an absolute value < 50%, (2) a > 15% relative reduction in global longitudinal strain (GLS), (3) a new rise in cardiac biomarkers including cardiac troponin, B-type natriuretic peptide (BNP), and N-terminal pro-BNP, or (4) heart failure symptoms^17)^. Therefore, a multimodal monitoring strategy using echocardiography (LVEF and GLS), cardiac biomarkers (cardiac troponin, BNP, and NT-proBNP), electrocardiography, and cardiac magnetic resonance imaging (CMR) as well as clinical assessment is recommended in all patients starting cancer therapy at baseline and during active cancer therapy as part of their cardiovascular risk assessment.

Several interventional studies and long-term observational cohorts revealed no reports of CTRCD for either hydroxyurea, pegylated IFN-α2a, ropeginterferon α-2b, or ruxolitinib^10-13)^. The cardiovascular events in these studies may primarily reflect the underlying thrombotic nature of PV rather than direct drug-induced myocardial injury.

We recently reported on left ventricular dysfunction related to one year of ropeginterferon α-2b therapy for PV^18)^. Screening echocardiography revealed a decline in LVEF to 46%. Late gadolinium enhancement CMR revealed slight midwall hyperenhancement in the inferior and lateral wall, suggesting non-ischemic myocardial injury. After discontinuing ropeginterferon α-2b, the LVEF rapidly recovered to 60%. However, it declined again to 46-51% upon rechallenge. This was a reversible, dose-dependent, IFN-related myocardial injury. This fluctuating course under stable hematologic control strongly suggests a causal relationship between ropeginterferon α-2b and cardiac dysfunction.

IFN is classified as type I (including IFN-α, which is used to treat PV), type II, or type III^19)^. Recent evidence indicates that type I IFN signaling via the JAK-STAT pathway can indirectly promote mitochondrial stress by inducing IFN-stimulated genes that alter cardiomyocyte protein translation, metabolic regulation, and cellular stress responses^19)^. Concurrently, type I IFN amplifies cyclic GMP- AMP synthase (cGAS)-stimulator of IFN genes (STING) activation triggered by mitochondrial DNA leakage, thereby exacerbating inflammatory signaling, impaired mitochondrial function, and cardiotoxicity. Together, these pathways can create a positive feedback loop that exacerbates mitochondrial dysfunction in stressed or diseased cardiomyocytes through IFN-dependent signaling^19)^. Our case highlights that long-term ropeginterferon α- 2b therapy for PV, although generally well tolerated^12)^, may cause CTRCD, requiring periodic cardiac monitoring^18)^.

### Monitoring and multidisciplinary care for cardiovascular risk in PV

Taken together, these clinical observations highlight the necessity of a structured, proactive cardiovascular management strategy for patients with PV. Despite the favorable safety profile of cytoreductive therapies for PV, the integration of cardio- oncology principles into routine care has become essential due to the high cardiovascular risk associated with PV and the possibility of CTRCD. Effective management should begin with a comprehensive baseline cardiovascular evaluation and be followed by risk-stratified, longitudinal monitoring that considers both disease-related and treatment- specific factors. Although early detection and management of CTRCD are ideal, further data are needed to determine the appropriate timing of cardiovascular follow-up for asymptomatic PV patients.

**Figure 1:**
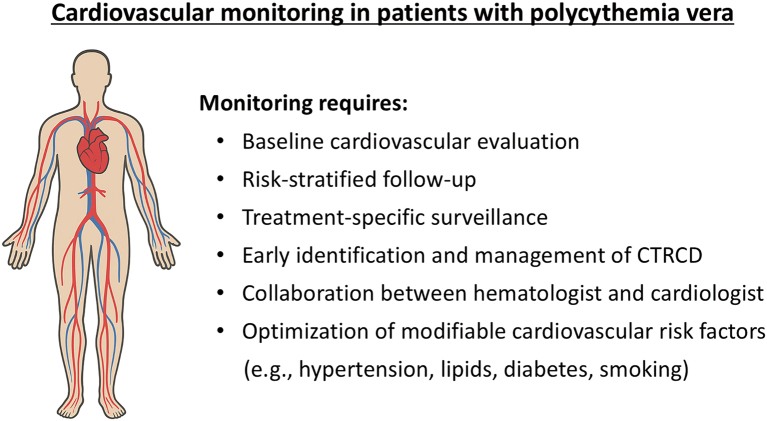
Cardiovascular monitoring in patients with PV CTRCD, cancer therapy-related cardiac dysfunction.

Achieving optimal outcomes also requires close collaboration among hematologists, cardiologists, and cardio-oncology specialists to ensure the timely interpretation of cardiovascular findings and coordinated therapeutic decision-making. In parallel, aggressively optimizing modifiable cardiovascular risk factors, including hypertension, dyslipidemia, diabetes, and smoking, remains a cornerstone of comprehensive cardiovascular prevention in PV (Figure 1).

## Conclusion

Cardiovascular disease plays a central role in the morbidity and mortality associated with PV. Although phlebotomy, aspirin, and cytoreductive therapy are important for reducing thromboembolic risk, attention must also be paid to treatment- related cardiotoxicity. Integrating the guideline- based cardio-oncology monitoring into routine PV management will improve safety and long-term outcomes. Further research is needed to clarify the mechanisms of IFN-associated cardiotoxicity.

## Author contributions

MM and TA equally contributed to the conception, design, collection, and interpretation of the data. The first draft of the manuscript was written by MM and TA. All authors read and critically revised the manuscript and approved the final manuscript.

## Conflicts of interest statement

Dr. Matsue received honoraria from Otsuka Pharmaceutical Co., Novartis Pharma K.K., Bayer Inc., and AstraZeneca, and research grants from Pfizer Japan Inc., Otsuka Pharmaceutical Co, EN Otsuka Pharmaceutical Co., Ltd., and Nippon Boehringer Ingelheim Co., Ltd. The other authors have no conflicts of interest to declare.
